# Research from low-income and middle-income countries will benefit global health and the physiotherapy profession, but it requires support

**DOI:** 10.4102/sajp.v79i1.1957

**Published:** 2023-09-22

**Authors:** Saurab Sharma, Arianne P. Verhagen, Mark Elkins, Jean-Michel Brismée, George D. Fulk, Jakub Taradaj, Lois Steen, Alan Jette, Ann Moore, Aimee V. Stewart, Barbara J. Hoogenboom, Anne Soderland, Michele Harms, Rafael Z. Pinto

**Affiliations:** 1 School of Health Sciences, Faculty of Medicine and Health, University of New South Wales, Sydney, Australia; 2 Centre for Pain IMPACT, Neuroscience Research Australia, Sydney, Australia; 3 International Society of Physiotherapy Journal Editors, Australia; 4 Journal of Physiotherapy, Australia; 5 Journal of Manual and Manipulative Therapy, United States; 6 Journal of Neurologic Physical Therapy, United States; 7 Physiotherapy Review, Poland; 8 Fysioterapi (Swedish Physiotherapy Journal), Sweden; 9 PTJ: Physical Therapy & Rehabilitation Journal, United States; 10 Musculoskeletal Science & Practice, United Kingdom; 11 South African Journal of Physiotherapy, South Africa; 12 International Journal of Sports Physical Therapy, United States; 13 European Journal of Physiotherapy, United Kingdom; 14 Physiotherapy, United Kindgom; 15 International Society of Physiotherapy Journal Editors and Brazilian Journal of Physical Therapy, Brazil

Disparities in research publications are common in the physiotherapy and rehabilitation fields (Sharma et al. 2019b). A small proportion of published research arises from low-income and middle-income countries (LMICs) (Sharma et al. 2019b; Tamrakar et al. 2021), home to 85% of the world’s population. Systems-level, institutional-level and individual-level factors contribute to these disparities. With urgent and unified actions, global health and the standard of physiotherapy research in LMICs can be improved and strengthened. In this editorial, we will discuss the challenges encountered by researchers from LMICs in conducting and publishing high-quality research and propose potential strategies to address these challenges.

## Background

LMICs are defined as countries with a cumulative annual gross national income per capita of US$13 205 or less (Table 1) (The World Bank 2022). Many LMICs have a higher prevalence of injuries and long-term conditions requiring rehabilitation services compared to high-income countries (Cieza et al. 2021; Vos et al. 2020). Despite the greater disability burden in LMICs, the quality and quantity of research conducted in these countries are underwhelming. For example, despite being ranked as the number one cause of disability, low back pain lacks primary data from many LMICs (Sharma & McAuley 2022; Tamrakar et al. 2021).

**TABLE 1:** Categorisation of low-, middle- and high-income countries based on the World Bank.

Income bracket	2021 gross national income per capita (USD)	Countries (*n*)	Example countries	Pooled population
Low	≤ $1085	28	Afghanistan, Ethiopia, Malawi, Liberia, Somalia, Sudan, Syria, Uganda, Yemen	0.7 billion
Lower-middle	$1086 – $4255	54	Bangladesh, Bolivia, Egypt, Haiti, India, Indonesia, Kenya, Nepal, Philippines, Tajikistan, Vietnam	3.3 billion
Upper-middle	$4256 – $13 205	54	Albania, Botswana, Brazil, China, Colombia, Jamaica, Jordan, Kazakhstan, Libya, Malaysia, Mexico, South Africa	2.5 billion
High	> $13 205†	80	Aruba, Australia, Belgium, Canada, Chile, France, Israel, Japan, Latvia, Malta, Poland, Qatar, Singapore, United Kingdom (UK), United States of America (USA)	1.2 billion

† , Gross national income of high-income countries ranges from US$ 13 260 for Seychelles to US$ 116 540 for Bermuda (UK) reflecting a wide range of countries included within this category.

LMICs have different research priorities than high-income countries. Compared to high-income countries, LMICs often have underdeveloped and resource-limited health systems and different disease burdens, with research only starting to catch up. Because of between-country/culture differences in illness beliefs and coping strategies, research from high-income countries (Sharma et al. 2020), where most evidence is generated, may not apply in LMICs. In other words, interventions developed using resources and clinical populations in high-income countries may not be culturally appropriate or feasible in LMICs. For example, education, a commonly recommended intervention for chronic health conditions, needs to consider patients’ socioeconomic and cultural factors (Sharma et al. 2019b). This warrants significant cultural adaptation and testing of healthcare interventions when used in different health settings/systems. Given that there is limited local research on many clinical conditions, clinicians in LMICs are left to rely on research from high-income countries for patient care. Although the need for high-quality research to address local research gaps has been frequently highlighted, research publications from the LMICs remain limited (Buchbinder et al. 2018).

## Barriers related to conducting high-quality research in LMICs

While LMICs may share some common barriers to conducting and publishing research with high-income countries, some challenges are unique to LMIC contexts (Conradie et al. 2018). Many LMICs do not prioritise research, which results in a lack of research funding, no research culture, limited awareness of research and no research workforce (Conradie et al. 2018). LMICs rarely include research funding in their national budgets. Awareness about research is lacking at all levels and therefore health professionals, academic staff and the general public have little to no understanding of research. School and university curricula also lack a research focus.

Academic institutions do not offer adequate research support to their staff and students, who lack research training, ongoing mentorship, research resources and infrastructure (including but not limited to access to online databases), reliable Internet access, secured computers and statistical software. Unfair expectations are imposed on academic staff to generate research productivity without dedicated research hours and administrative support (e.g. research assistants). For example, publishing two or more original research papers is commonly expected in three years for academic promotion. In a survey of researchers from 27 African countries, lack of dedicated research-related roles was the most common barrier identified, reported by 48% of the respondents (Conradie et al. 2018).

## Barriers/threats to publishing research from LMICs

Barriers to publishing research are linked with the barriers to conducting research described above. One widely known barrier is language. Writing academic papers can be daunting, especially when writing in a non-native language. Researchers in LMICs who do not speak English as their first language find it especially challenging to write journal articles in English. They also lack local support to improve their writing skills.

The second barrier is related to, as alluded to earlier, the differences in research priorities in LMICs and high-income countries. Many international journals lack geographic diversity in their editorial boards. As a result, the manuscript handling editors (and reviewers) lack adequate understanding of the local research contexts and the need for studies in LMICs when looking from their own lenses of research priorities in high-income countries. Finding reviewers who understand local research contexts is also challenging, further complicating the editorial decision.

Third, researchers in LMICs are frequently the targets of predatory publishers. As a consequence, researchers from LMICs publish their work in ‘predatory’ journals (Shen & Bjork 2015). The researchers see predatory journals as ‘low-hanging fruit’, despite costs associated with publication, because the publishing requirements are easier to meet while the publication process is often swift, in contrast to (international) peer-reviewed reputable journals.

Lastly, some editors/reviewers of international peer-reviewed journals deem research from LMICs to have local relevance and impact only, and therefore flag them as more appropriate for local journals. The major problem with this is that most local journals are not indexed and are therefore often undiscoverable through traditional databases (e.g. PubMed). Publishing in these non-indexed local journals contribute to duplicate research and therefore research waste (Ioannidis et al. 2014; Sharma et al. 2019a). For example, 75% of research on clinical pain in Nepal was published in local journals with duplicate and redundant research (Sharma et al. 2019a). The international research community should facilitate research from LMICs so that this research can make both local and international impacts.

## A call to action

Urgent actions can help address key barriers to conducting and publishing research in LMICs. The potential solutions to each of the barriers are presented in Table 2.

**TABLE 2:** Barriers to research in low- and middle-income countries (LMICs) and proposed solutions.

Type of barrier	Barriers	Proposed solutions
Barriers to conducting research	Lack of research priority	Government and academic institutions should prioritise research and develop a research priority agenda for common health conditions. Funding should be allocated towards addressing these research priorities.
	Research awareness and education	Research should be introduced early in school and during undergraduate education. The importance of research should be shared with the public. Academic institutions and clinical settings (e.g., hospitals) should emphasise the importance of both conducting and using research for improving healthcare.
	Research funding	Governments and universities should allocate a defined proportion of their budgets for research. Research scholarships should be awarded for postgraduate degree and postdoctoral fellowships.
	Lack of institutional support	Universities should support researchers. Full-time and part-time research roles should be created so that researchers can commit their time and focus on conducting high-quality research. Academic staff should be allocated research hours to allow dedicated research time. Institutions should also favourably appraise high-quality research over any research.
	Lack of research workforce	As above, both government and universities should support full-time and part-time research roles to develop research workforce and high-quality research skills.
	Lack of research training	Government and universities should promote high-quality research training. They should also promote local and international research mentoring as well as collaborations across fields.
Barriers to publishing research	High editorial bar for international journals	Journals could offer special editorial support to authors from LMICs. International collaborations or mentorship could help with the publication process. Appointing editors and reviewers who understand local research priorities and contexts and who are better able to make informed judgement about the need for the research and its potential impact.
	Language barrier	Proofreading of the final manuscripts by native English-speaking collaborators and journal editorial board members could address language barriers related to publishing in an international journal. AuthorAID (https://www.authoraid.info/en/) connects researchers from LMICs to the international community. Mentors and mentees can connect through the AuthorAID programme.
	Predatory publishing	Frequent webinars around publishing in the right journals should be offered. Government, funding bodies (if present), and institutions should emphasise publishing in credible journals. Authors should think critically about the credibility of the journals they submit to, drawing on resources like Think, Check and Submit (http://thinkchecksubmit.org).

### What can researchers from LMICs do?

Early career researchers may initiate collaborations with experienced local and international researchers with shared interests. Experienced researchers may extend mentorship opportunities to junior researchers both locally and internationally, facilitate inclusion on a journal editorial board and volunteer to review papers.

### What can international journals do?

International physiotherapy journals are in a strong position to support and promote physiotherapy research in LMICs, especially through the leadership of the *International Society of Physiotherapy Journal Editors*.

First, journals should consider equity, diversity and inclusion within the editorial board – not only in terms of gender and race but also diversity based on national economies. Editors who understand local research contexts are better able to make informed editorial decisions. Where possible within the scope of the journal, editorial decisions should be made considering research priorities from the originating country and the impact of the research there. This will assist with providing strong research foundations for LMICs.

Second, editors should also prioritise recruitment of reviewers with research experience in LMICs, preferably from the same countries where the research was conducted. Editors may also request specific feedback regarding whether the study methods are appropriate for the local context.

Third, journals may provide additional support to authors from LMICs. This may include providing additional assistance in editing, proofreading or responding to peer reviewers. Journal websites may provide specific guidance to authors with limited publishing experience, such as links to resources for conducting and writing research. Journals may also offer current and prospective authors Massive Open Online Courses (MOOCs) on research and academic writing, with the content targeted at authors from LMICs. Alternatively, paid workshops might be offered with a waiver for participants from LMICs.

**FIGURE 1:**
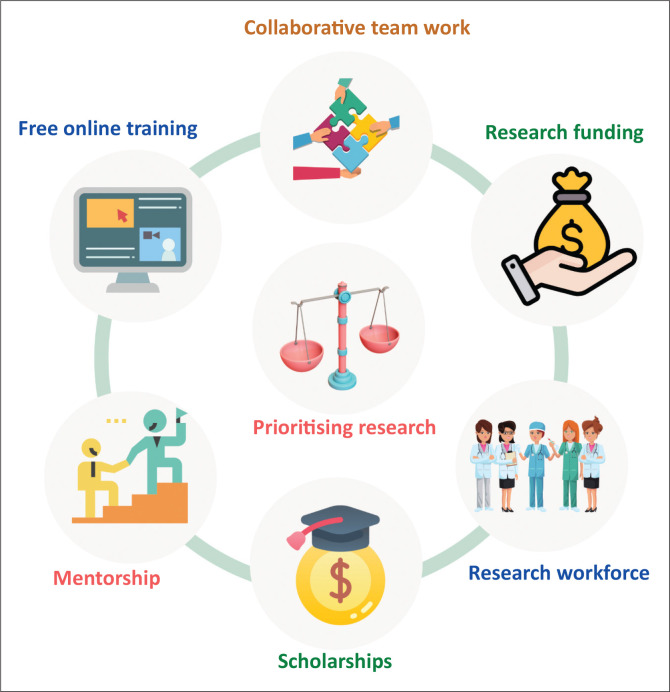
Strategies to promote high-quality research in low- and middle-income countries.

A final strategy to consider is that publishers/journals may guide authors who need mentorship to a list of volunteer mentors. The journal manuscript submission platform could include a button to click if the author is looking for a mentor or is willing to mentor. The extent of mentorship could vary from proofreading the current paper, to analysing data, to assisting in writing a paper, to mentoring the development of a research question. The latter can especially be valuable for many, as journal editors frequently encounter research with poorly developed research questions and flawed research methods. With these problems, even the most supportive editors cannot help as it is too late to help. One initiative to support early career researchers is available through AuthorAID (https://www.authoraid.info/en/). Authors may choose to be listed as a mentor or a mentee and provide or receive mentorship at various stages of research including planning, analysis, writing, editing and proofreading. Strategies to promote high-quality research in LMICs are summarised in Figure 1.

## ‘Bright spot’

Despite several significant challenges, research with wide-scale implications has started to arise from LMICs which addresses local health research priorities (Guimarães et al. 2021; Liang et al. 2022; Shah et al. 2022; Sharma et al. 2019b). Some prominent journals in physiotherapy, pain, science and medicine have already started to identify the importance of equity, diversity and inclusion in health research which is likely to make a meaningful impact in promoting research from LMICs (McCambridge & Elkins 2021; Palermo et al. 2023; The Editors 2021; The Editors of The Lancet Group 2019; Nature Medicine 2021). Selected publishers also waive publication fees for authors from LMICs to assist in open-access publishing.

## Conclusion

Local research in LMICs is necessary to advance science and improve patient care in these settings. However, researchers in LMICs face several unique challenges to conduct and publish their research internationally. The *International Society of Physiotherapy Journal Editors* and member journals support research from the LMICs to improve the health and lives of the 85% of the world’s population that lives in LMICs.
